# A comprehensive diagnostic approach combining phylogenetic disease bracketing and CT imaging reveals osteomyelitis in a *Tyrannosaurus rex*

**DOI:** 10.1038/s41598-020-75731-0

**Published:** 2020-11-03

**Authors:** C. A. Hamm, O. Hampe, D. Schwarz, F. Witzmann, P. J. Makovicky, C. A. Brochu, R. Reiter, P. Asbach

**Affiliations:** 1 Department of Radiology, Charité—Universitätsmedizin Berlin, corporate member of Freie Universität Berlin, Humboldt-Universität zu Berlin and Berlin Institute of Health, Charitéplatz 1, 10117 Berlin, Germany; 2 Institute for Diagnostic Radiology and Neuroradiology, Greifswald University Hospital, Ferdinand-Sauerbruch-Straße, 17475 Greifswald, Germany; 3 Museum für Naturkunde, Leibniz-Institut für Evolutions- und Biodiversitätsforschung, Invalidenstraße 43, 10115 Berlin, Germany; 4 Department of Earth and Environmental Sciences, University of Minnesota, Minneapolis, MN 55455 USA; 5 Field Museum of Natural History, 1400 S. Lake Shore Dr, Chicago, IL 60605 USA; 6 Department of Earth and Environmental Sciences, University of Iowa, Iowa City, IA 52242 USA; 7 Richard and Loan Hill Department of Bioengineering, University of Illinois, Chicago, IL 60607 USA

**Keywords:** X-ray tomography, Bacterial infection, Disease model, Palaeontology

## Abstract

Traditional palaeontological techniques of disease characterisation are limited to the analysis of osseous fossils, requiring several lines of evidence to support diagnoses. This study presents a novel stepwise concept for comprehensive diagnosis of pathologies in fossils by computed tomography imaging for morphological assessment combined with likelihood estimation based on systematic phylogenetic disease bracketing. This approach was applied to characterise pathologies of the left fibula and fused caudal vertebrae of the non-avian dinosaur *Tyrannosaurus rex*. Initial morphological assessment narrowed the differential diagnosis to neoplasia or infection. Subsequent data review from phylogenetically closely related species at the clade level revealed neoplasia rates as low as 3.1% and 1.8%, while infectious-disease rates were 32.0% and 53.9% in extant dinosaurs (birds) and non-avian reptiles, respectively. Furthermore, the survey of literature revealed that within the phylogenetic disease bracket the oldest case of bone infection (osteomyelitis) was identified in the mandible of a 275-million-year-old captorhinid eureptile *Labidosaurus*. These findings demonstrate low probability of a neoplastic aetiology of the examined pathologies in the *Tyrannosaurus rex* and in turn, suggest that they correspond to multiple foci of osteomyelitis.

## Introduction

In the early twentieth century, Roy Lee Moodie, a geologist and palaeontologist, established the field of palaeopathology, the study of diseases and traumatic injuries that caused visible abnormalities of the skeleton in fossil vertebrates^1^. Studying pathological phenomena provides insights into the life of extinct animals, their behaviour, growth, interactions and healing processes. Moreover, understanding the origin and evolution of palaeopathologically diagnosable diseases provides important insights into the evolution of present-day diseases in veterinary and human medicine^1^. Understanding the cause and propagation of animal diseases is important, as zoonotic pathogens are related to over 75% of all human diseases^2^. Evidence supporting a diagnosis in extinct species is based largely on fossilised bones and teeth, as other structures are only rarely preserved. Many dinosaurian pathologies have been documented^3^. However, owing to the scarcity of detailed descriptions of palaeopathological case studies and the low prevalance of bone pathologies in any species in general the diagnosis of skeletal abnormalities among theropod dinosaurs remains challenging^1^.

Interdisciplinary collaborations can help overcome many of these limitations and improve palaeopathological diagnoses of skeletal abnormalities^4,5^. In this context, radiological imaging is a substantial asset in palaeopathology as it aids in visualising internal structures and reveals many additional diagnostic features, thus bringing to light hallmarks of abnormalities also seen in human and veterinary medicine^6–8^. However, these may only narrow down the differential diagnosis rather than providing a definite diagnosis. Moreover, the morphological features presented by a particular disease can vary among species, which calls for a multistep approach in diagnosing pathological lesions in the fossil record^6,9^.

In this test case, the Extant Phylogenetic Bracket (EPB)^10,11^ was applied to derive further information from previous pathology reports and epidemiological analyses comparing different species. Consideration of the prevalence of a disease in the species of interest and its close extant relatives (narrow phylogenetic disease bracket, NPDB) limits the number of possible differential diagnoses^12–15^. Such data can contribute substantially to more robust and reliable findings.

*Tyrannosaurus rex*, a Late Cretaceous North American theropod, is among the largest predatory dinosaurs^16–18^ and is probably the most famous non-avian dinosaur (NAD). It remains a subject of interest in vertebrate palaeontology, as documented by recent articles about the evolution of its giant body and advanced senses^19,20^, facial sensory system^21^, bite force^22^, feather evolution^23^ and locomotive capabilities^24^, and also about its systematic relationships and evolutionary history^25^. *Tyrannosaurus rex* has been reported as presenting with a variety of medical diagnoses, including a number of pathologies observed in the individual studied here^18,26,27^. These pathologies were based upon earlier diagnoses using gross morphology alone, or in combination with computed-tomography (CT) imaging^26,28^. However, phylogenetic evidence and disease prevalence were not systematically taken into account^26,29–33^.

Therefore, the purpose of this study was to establish a comprehensive concept for thorough palaeopathological diagnosis based on a combination of detailed radiological lesion characterisation^6–8^ and epidemiological likelihood estimation by phylogenetic disease bracketing including birds and non-avian extant reptiles (crocodylians, lepidosaurs, testudines) as members of this bracket. This concept was applied to diagnose the pathologies observed in the caudal vertebrae and left fibula of the *Tyrannosaurus rex* specimen FMNH PR2081.

## Results

### Description of pathology (internal and external morphology)

**Figure 1:**
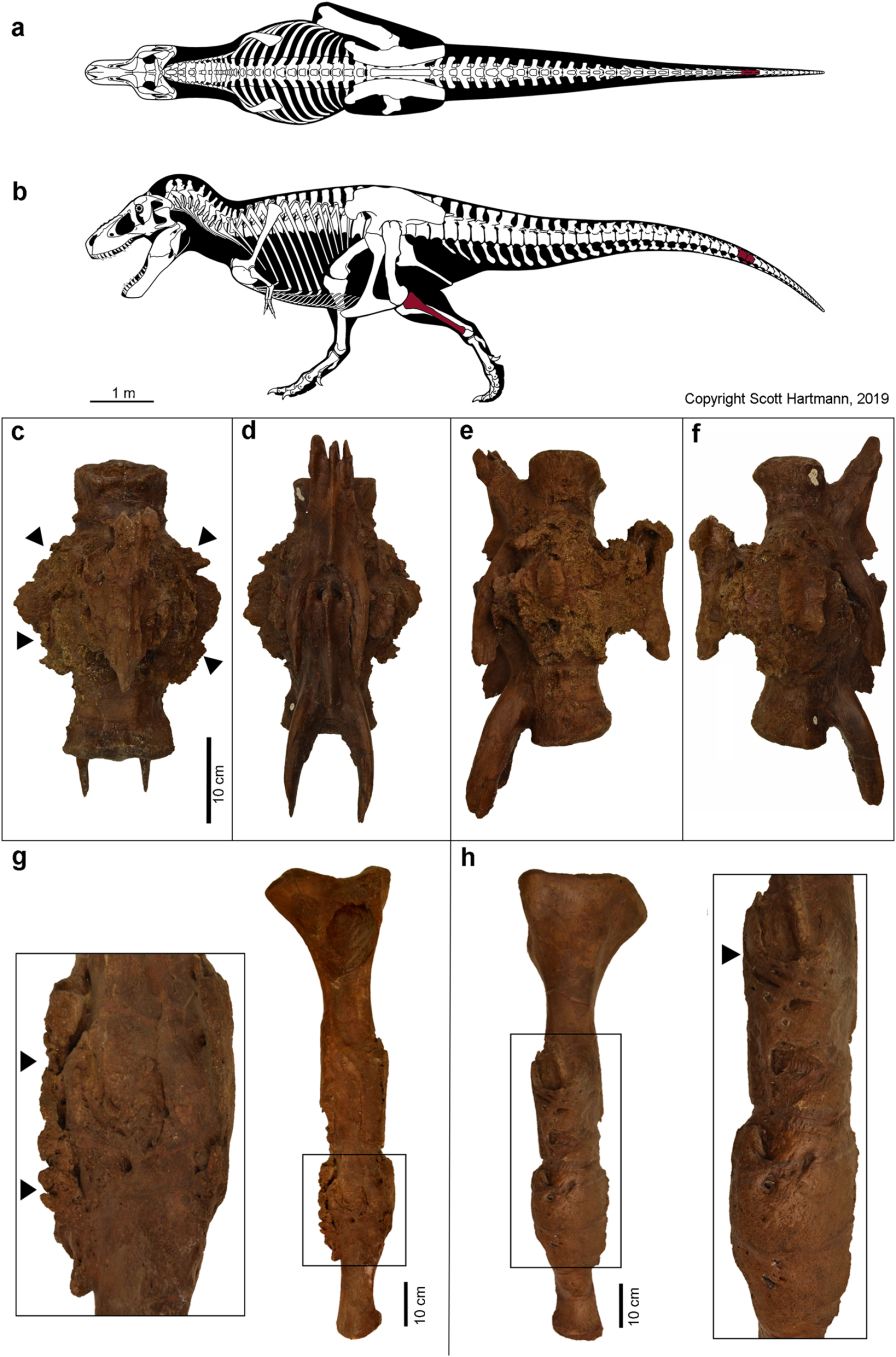
*Tyrannosaurus rex* specimen FMNH PR2081 and gross appearance of the fused caudals and left fibula. (**a**,**b**) Illustration of the articulated skeleton of the *Tyrannosaurus rex* ‘Sue’. The bones under investigation in this study, the left fibula and fused caudal vertebrae c26 and c27, are highlighted in red. (**c**–**f**) The readily apparent circumferential rugose bone formation of the fused caudals is indicated by arrowheads. (**g**,**h**) Fibula with magnification of pathological changes; arrowheads indicate the rugose surface. (Copyright Scott Hartman, 2019. Modified and used with permission).

The pathologies of the fibula and the fused caudal vertebrae are readily apparent on gross examination (Fig. 1). The detailed lesion description is based on gross examination and radiological cross-sectional imaging by CT.

#### Left fibula

##### Gross examination

**Figure 2:**
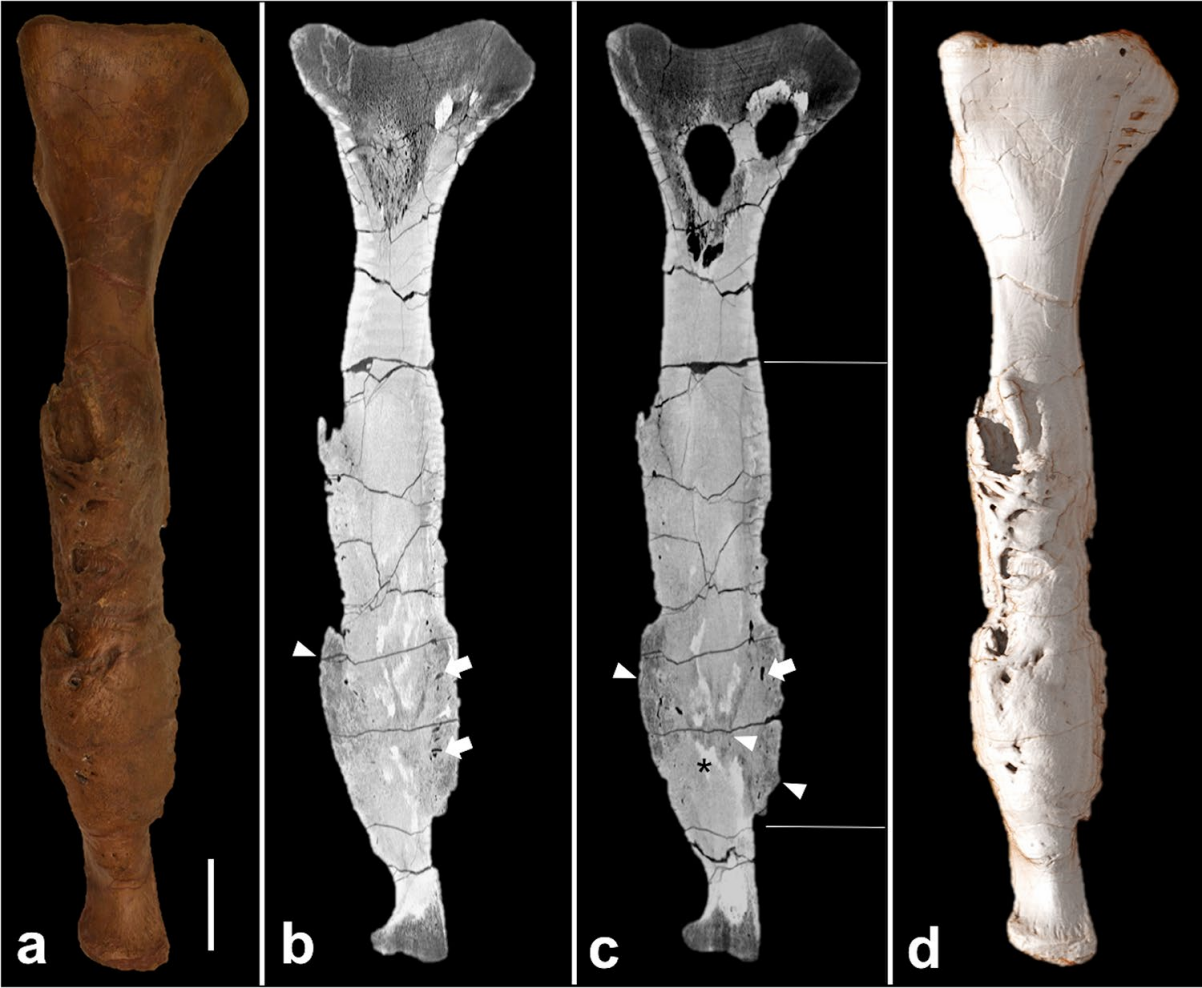
Fibula. (**a**) Gross appearance; (**b**,**c**) sagittal cross-sectional CT images; (**d**) 3D reconstruction based on CT images. The sagittal cross-sectional CT images demonstrate (i) increased bone thickness, limited to the two horizontal white bars; (ii) decreased bone density in the area of the active focus of the infection (arrowheads); (iii) tubular canals in the periosteal bone formation (arrows). The scale bar represents 10 cm.

The fibula shows an altered bone structure in the distal two-thirds of the shaft compared with the normal osteological condition present on the right side. The proximal and distal articular surfaces are not affected and do not show any signs of pathological changes. The pathologically thickened and altered bone is approximately 68 cm long, becoming decreasingly distinguishable until it converges completely with adjacent normal bone. The remodelled bone surface of the pathological lesion has several recesses and is irregular compared with the smooth periosteal surface on the right fibula. The overall axis of the bone is not altered and, compared with the opposite fibula, the bone is not shortened (Figs. 1, 2).

##### Cross-sectional CT imaging

**Figure 3:**
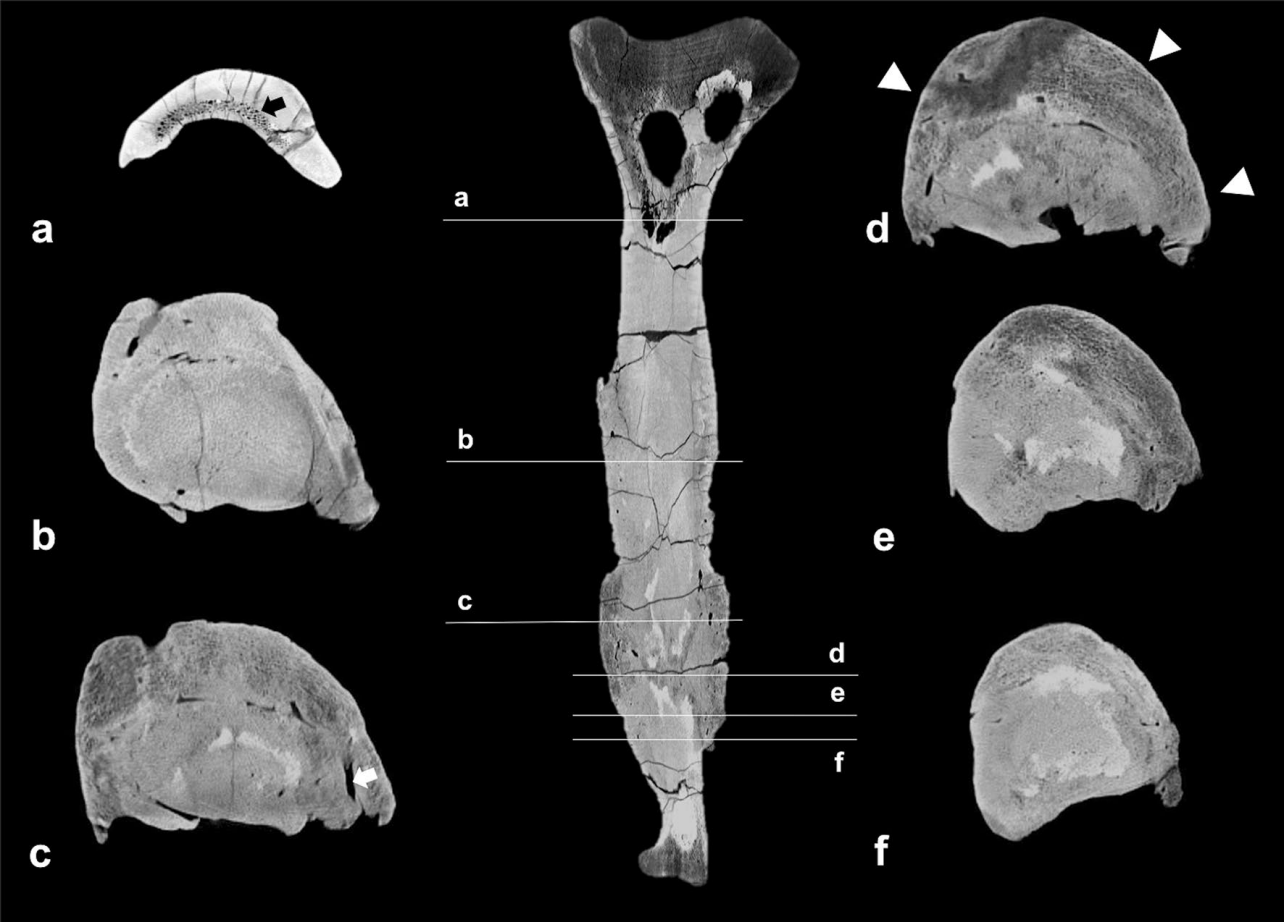
Cross-sectional CT images of the fibula at different levels of the bone, demonstrating the changes of bone architecture throughout the bone. Arrowheads indicate the pronounced periosteal bone formation. The white arrow indicates one of the tubular canals of the periosteal bone formation. The black arrow indicates the trabecular bone in the proximal part of the fibula.

The proximal third and the most distal part of the fibula have well-preserved cortical and trabecular bone structures. Extensive periosteal bone formation is in the distal two-thirds, except for the distal epiphysis. The periosteal mass-like bone formation obscures the adjacent non-pathological bone cortex and cannot be delineated precisely, although the outline between the original bone and periosteal bone formation is discernible (Fig. 3). The newly formed bone reveals both hyper- and hypodense areas, which partly contain sediment enclosures. In the distal third, the trabecular bone is replaced by more hypodense bone structure and the internal structure of the mass is characterised by small diffuse hypodense areas (Figs. 2, 3) compared with the proximal portions of the diseased bone. However, no evidence of diffuse or focal lytic areas is seen. The circumferential periosteal bone formation has several tubular structures, mostly limited to the newly formed bone and not extending into the central part of the non-pathological bone.

#### Caudals

##### Gross examination

**Figure 4:**
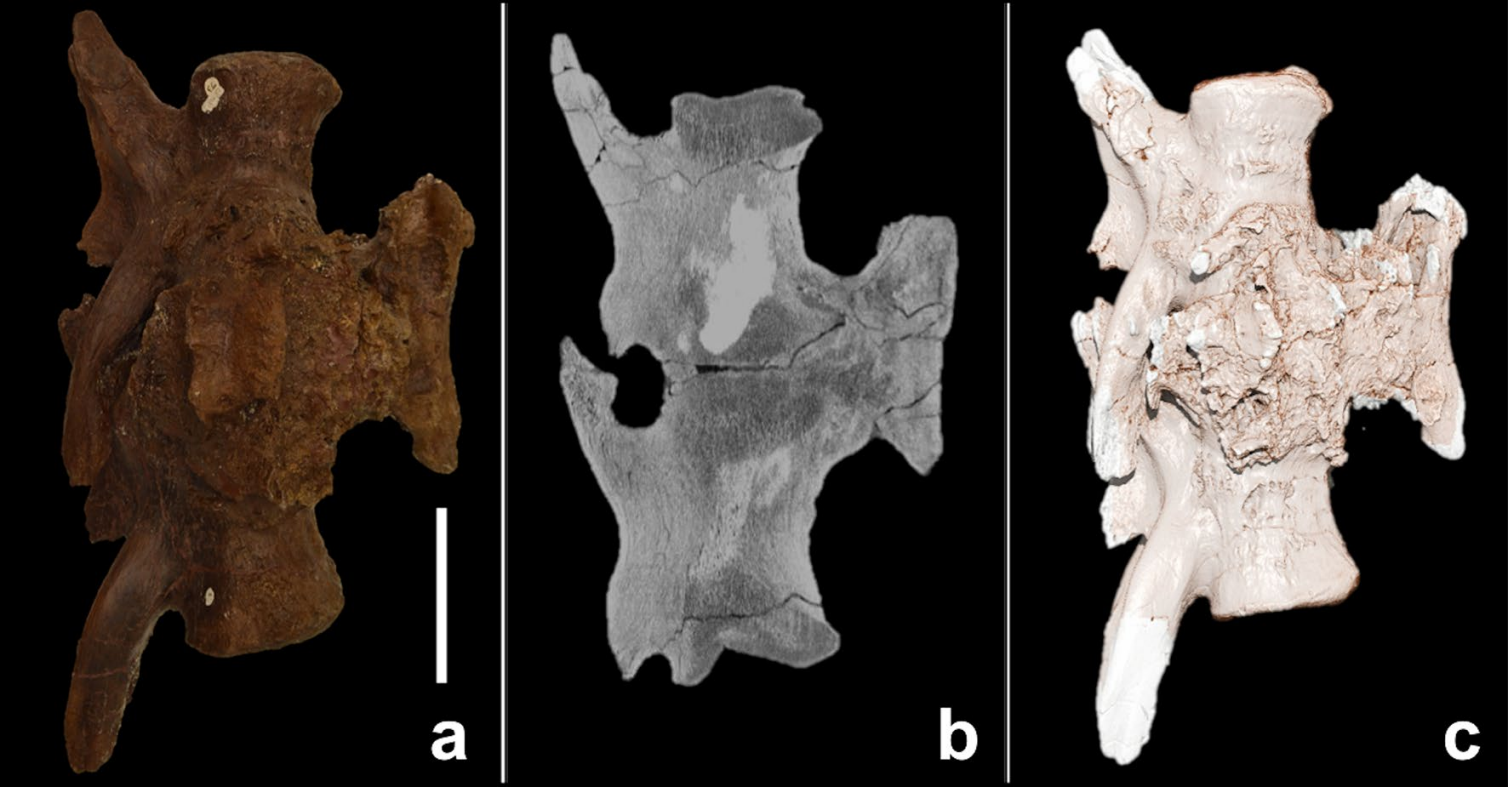
Fused caudals. (**a**) Gross appearance; (**b**) sagittal cross-sectional CT image; (**c**) 3D reconstruction based on CT images. The scale bar represents 10 cm.

The caudal vertebrae c26 and c27 and their mutual haemal arch are fused together. The vertebrae show no signs of deformation or shortening, as supported by cross-sectional imaging. The pathology presents as a diffuse, mass-like enlargement at the level of the intervertebral articulation and has a total rostral-caudal length of 14.5 cm and a diameter of 19.8 cm. The periosteal bone formation extends from the intervertebral fusion zone to the haemal arch and appears rugose and irregular apart from long, bilaterally symmetric rostral-caudal grooves. These grooves have been described before and probably served as attachment areas for the ventral tail musculature^26^. The zygapophyseal joints are not affected and no signs of tendon ossification are seen (Figs. 1, 4).

##### Cross-sectional CT imaging

**Figure 5:**
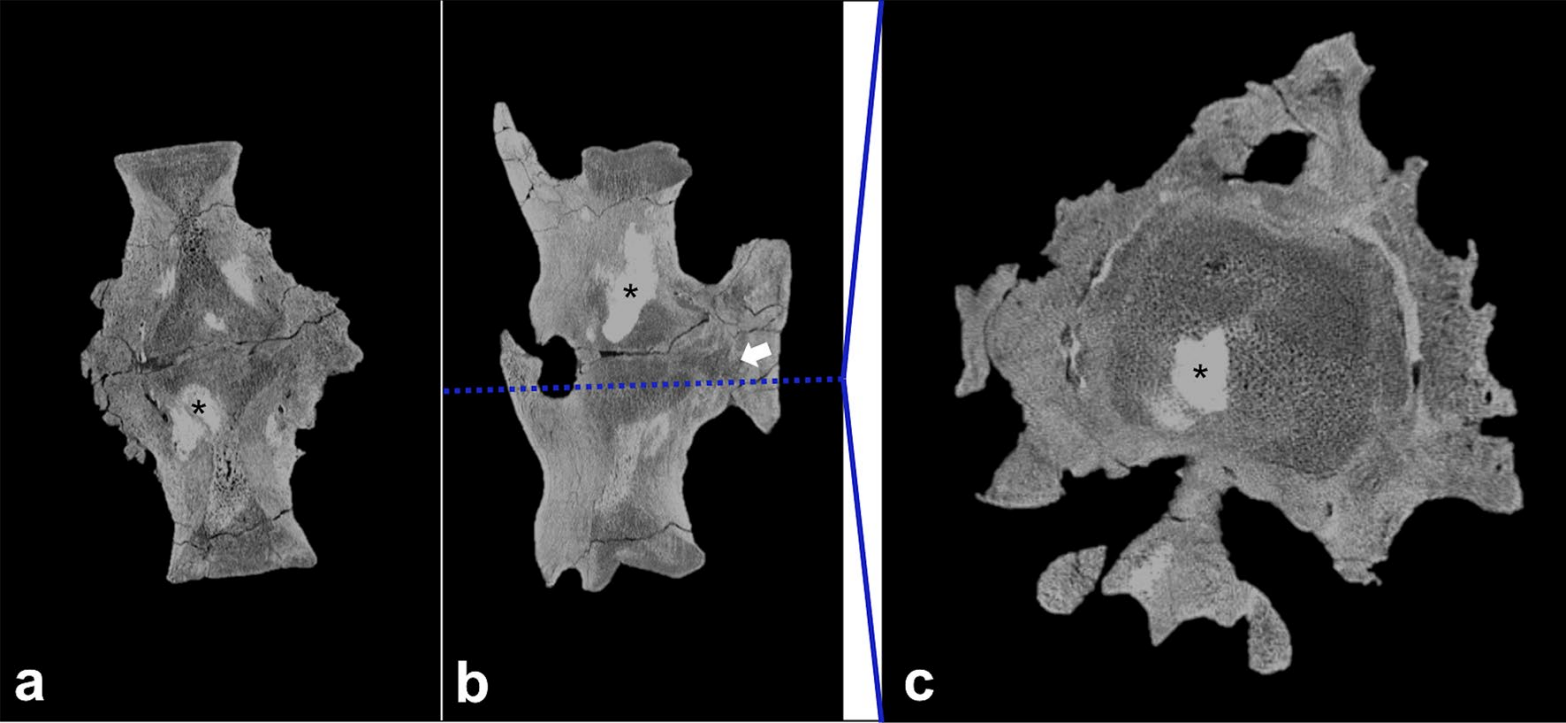
Cross-sectional images of fused caudals. The pathological changes are recognisable on the coronal (**a**), sagittal (**b**) and axial (**c**) plane. The axial plane demonstrates the cross-section of the bone on the level of the blue line, indicated in the sagittal plane. The black asterisks (*) indicates areas with increased density within the lesion, presumably due to sediment enclosures; the white arrow indicates decreased bone density in the area between the haemal arch and intervertebral articulation, suggesting an active focus of infection.

The rostral and caudal articular surfaces of the fused caudal mass appear normal and continue with a regular trabecular and cortical bone pattern for approximately 5 cm and 6.5 cm in a rostral and caudal direction, respectively. Although the vertebrae are fused, the articular surfaces can be discerned in the scans (Fig. 5). However, compared with the healthy articular surfaces they are irregular and more flattened than concave. The underlying trabecular bone appears porous, with a blurred trabecular pattern, while slightly enlarged. The cortex is almost completely replaced by extensive new bone formation and remodelling. The periosteal bone formation is more hyperdense than the trabecular bone, but less dense than the cortical bone, and is up to 4.5 cm thick. The periosteal bone formation, extending from the intervertebral fusion zone to the haemal arch, shows centrally porous and hypodense regions (Fig. 5). This area is confined to the region between the articular surface and the haemal arch and is surrounded by extensive periosteal bone formation. Within the transition zone of rather hypodense to newly formed hyperdense bone, several tubular tracts can be seen, though not as many as seen in the left fibula. In the more hypodense areas of the bone, hyperdense sediment enclosures are present (Fig. 5). No large lytic areas are present within the abnormal bone.

### Differential diagnosis based on morphology

Bone alterations that occurred after the death of the animal, during the fossilisation process (such as breakage or plastic deformation) may mimic bone pathologies and should also be included in a differential diagnosis. However, the presence of new bone formation in the fibula and fused caudals under study clearly rules out pseudopathologies and indicates the presence of true pathological lesions^7,28,34^. Many different types of pathological osseous lesions are described in the literature. In general, lesions can demonstrate infectious/inflammatory, neoplastic, congenital, or metabolic characteristics and are commonly seen in clinical settings^35–41^. As described above, the diseased bones show the following main abnormalities: (i) diffuse periosteal bone formation with internal tubular structures, (ii) irregular, partly indistinguishable demarcation between newly-formed bone and non-pathological bone cortex, (iii) varying bone densities and trabecular bone structure within the pathological lesion, and (iv) fusion of two vertebrae and their haemal arch with a preserved intervertebral articular space. On the basis of these radiological and morphological characteristics we excluded metabolic or congenital processes as the cause of disease. Metabolic diseases, relevant in the fossil and veterinary record, primarily include osteomalacia, gout and Paget’s disease. Those conditions typically lead to rubbery bone texture and to deformities and erosive lesions within the joints (in osteomalacia^37,42^ and gout^7,37,42,43^) or to thickened outer bone cortex and a decreased number of thickened bone trabeculae (in Paget’s disease^44^). Congenital malformations are characterised rather by distorted frame structure and altered bone configuration, than by reactive bone formation and varying bone densities seen in the pathological lesions in this study^45,46^. The specimen FMNH PR2081 does not demonstrate any of the latter characteristics.

The fibula under investigation has been mentioned in previous studies; these suggested a chronic infection possibly in combination with a partially healed fracture, yet the origin of the pathology was not found^26,28,47^. Fractures are the most common bone pathologies in the fossil and veterinary record (see below); however, the bones under investigation do not show well-defined callus formation, as is present in some fractured ribs of FMNH PR2081^26,28^, and the overall axes of the bones are unaffected. In accordance with an earlier study, the preserved gap between the centra and the (probably intact) intervertebral space of the fused caudal vertebrae of FMNH PR2081 has been considered as an argument against the hypothesis that the cause of this abnormality was a fracture^26^. Therefore, the bones under investigation do not show convincing signs of recent or healed fractures. For the fused caudal vertebrae, diseases such as osteoarthritis, spondyloarthropathy and diffuse idiopathic skeletal hyperostosis (DISH) are considered in the differential diagnoses. Although the vertebrae are fused and present bony overgrowths, the pathology does not show typical signs of tendon or ligament ossification, subchondral erosive lesions/cysts, increased bone density under the articular surface (subchondral region) or zygapophyseal joint involvement^5,7,37,42,48,49^. In addition, the vertebral pathology presents as monosegmental joint involvement, which is rather uncommon in DISH and degenerative diseases^7,37^. Therefore, the vertebral pathology in this study does not fulfil diagnostic criteria for the latter differential diagnoses, so we exclude degenerative diseases. A neoplastic or infectious cause appears much more likely. Both can present as a multifocal mass with an invasive or non-invasive growth pattern, and both have been described in the human, NAD and veterinary literature (see below). However, a benign osseous neoplasm seems unlikely, given the finding of diffuse osseous thickening most consistent with a non-focal entity. Furthermore, a sharp demarcation of the lesion, commonly seen among benign lesions, is absent. Malignant or metastatic lesions typically grow in an infiltrative manner and destroy the architecture of the bone. The pathological lesions under investigation, which are similar in appearance and morphology, could be the result of a multifocal primary osseous malignancy, while the extensive new bone formation on the vertebral bones, fusing the haemal arch with the two vertebrae, could be interpreted as an aggressive and invasive growth pattern. A malignant primary bone tumour known to affect vertebral joints and long bones is chondrosarcoma^50–52^. This malignant neoplasm, originating from the trabecular region, generates cortical bone expansion, is oval or round in appearance and is associated with cortical thinning^7^. Yet the overall architecture of the intervertebral joint space and the configuration of the affected bones are unaltered, the pathological lesions do not originate in the trabecular region, the new bone formation is strictly confined to periosteal bone formation, and the general proportions of trabecular and cortical bone structures can be recognised in radiological cross sections. Also, malignant lesions typically show large lytic areas of destroyed bone and additional bone thickening^4,7,38,53–55^. Therefore, the pathological lesions in this study show features mostly inconsistent with typical malignant neoplasms, and specifically with chondrosarcoma. Nevertheless, a multifocal primary osseous malignancy remains a differential diagnosis worthy of further consideration.

As opposed to typical malignant neoplasms, osseous infections are characterised by a heterogeneity of features including (sub)periosteal bone reaction with irregular bone growth, disorganised architecture and intervertebral joint involvement^56^. Infections that affect the periosteum of the bone often lead to new bone formation and potential drainage or fistula formation^57,58^. The marked enlargement with irregular surface texture and increased thickness of large areas of the affected bones are morphologically most compatible with an infectious entity, where inflammatory cells and debris-containing fibrinous exudate accumulate; these are referred to as fibriscess in non-avian reptiles (NARs) and extant dinosaurs (birds)^59^. Moreover, the irregular and woven bone surface texture of the left fibula and fused vertebrae has been identified as a pathognomonic sign for non-specific osteomyelitis^7,60^. With regard to the morphological and imaging characteristics discussed above, osteomyelitis of the left fibula and fused caudal vertebrae is the most likely diagnosis.

### Differential diagnosis based on phylogenetic disease bracketing

#### Extant dinosaurs—birds

Studies on avian diseases do not distinguish strictly between captive and wild birds^38^. Traumatic events constitute the majority of bone abnormalities, as 86% of osteological disorders appear to be traumatic^61^. However, as outlined above, the left fibula and the fused caudal vertebrae of the specimen FMNH PR2081 show no evidence of an acute traumatic cause. Considering the enlargement of the surrounding bone, a chronic process after a focal traumatic event cannot be excluded at this point, especially as traumata constitute a common cause of secondary infectious processes within the bone^38,62^. Various metabolic diseases are described in avian medicine, the majority of which present as generalised conditions^38,62–64^. FMNH PR2081 does not demonstrate either the features of a generalised disease or the morphological features of the rare finding of focal osteoporosis^38^. In addition, joint-affecting gout is rare in birds compared with humans and is usually manifested as crystal deposition in visceral organs^28^. Therefore, a metabolic disease can be excluded as potential cause of the abnormalities of the left fibula and fused caudal vertebrae.

Since an acute traumatic event and an underlying metabolic cause both appear unlikely, the two remaining possible aetiologies are either neoplastic or infectious. The overall avian neoplasm rate in the literature analysis was 2.3% (3298/144,277 birds; Supplementary Table S1) indicating that neoplasms are rare among birds, as also suggested by others^65–67^. Although neoplastic diseases are not well studied among birds, the following, though rare^62,63,68^, primary bone tumours have been described: osteoma^68–70^, osteosarcoma^68,71^, chondroma^68^ and chondrosarcoma^64,68^. It has been reported that neoplasms are frequently seen in captive birds, yet the majority of these neoplasms were benign, and all were diagnosed as lipomas^72^. On a morphological basis we can exclude osteosarcoma and chondrosarcoma as potential differential diagnoses at this point, on the basis of the lack of malignant and invasive characteristics (see above). However, the two pathological lesions share the majority of morphological features and their multifocal appearance makes a disseminated osseous malignancy a relevant differential diagnosis. The benign features and the extreme rarity among birds makes osteoma a rather unlikely differential diagnosis^67,69,70,73^. The lack of articular proximity and the circumferential involvement of the bone make the differential diagnosis of an osteochondroma of the left fibula also highly unlikely, whereas it remains a differential diagnosis for the pathologically altered vertebrae.

**Figure 6:**
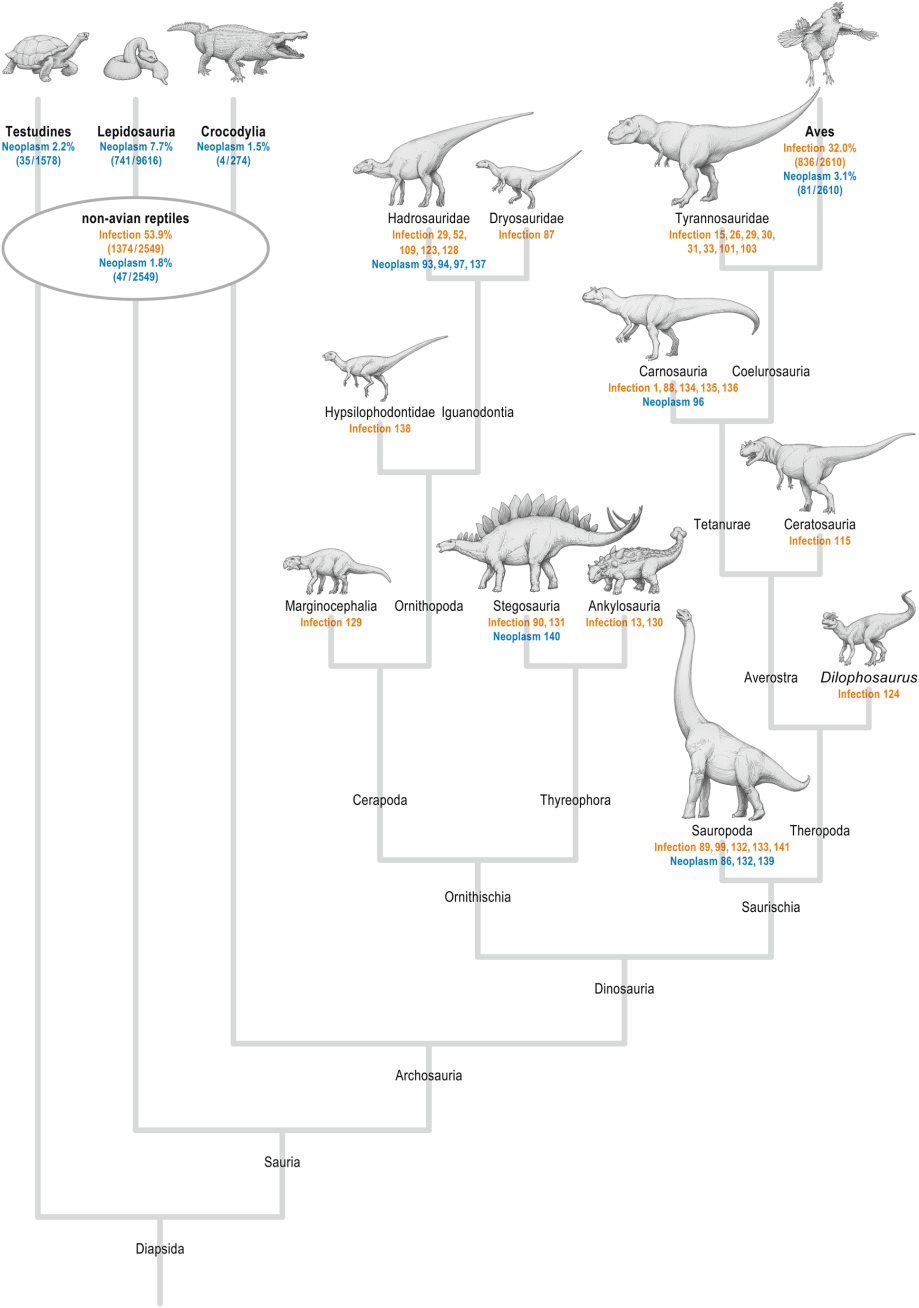
Neoplastic and infectious diseases in the dinosaur cladogram. Simplified non-avian dinosaur family tree showing the distribution of infectious (orange) and neoplastic diseases (blue) as derived from the literature analysis (numbers indicate the respective references). For Aves and non-avian reptiles, the prevalences of the respective diseases are given as percentages and derived from the pooled literature analysis given in Tables 1 and 2. The specific neoplastic disease rate for the non-avian reptile taxons testudines, lepidosaurs and crocodylians are derived from the pooled literature analysis given in the Supplementary Table 2^1,13,15,26,29–31,33,52,86–90,93,94,96,97,99,101,103,109,115,123,124,128–141^.

The pooled literature data on avian neoplastic and infectious disease prevalence revealed a distinctly higher rate for infections (32.0%, 836/2610 birds) than for neoplasms (3.1%, 81/2610 birds; Table 1, Fig. 6), suggesting that infection is much more likely. With bacterial infections being the most commonly diagnosed conditions among birds^64^ (Table 1) and the typical features of bone infection shown by the abnormalities under investigation here, the likeliest differential diagnosis is osteomyelitis.

**Table 1:** Prevalence of infectious and neoplastic disease in birds.

References	Wild (w) or captive (c)	Cohort size	Neoplasm	Infection		
				Total	Bacterial	Non-bacterial
Baker^142^	w	132	1	44	25	19
Halliwell^143^	c	125	2	80	17	63
Jennings^144^	w	112	0	53	22	31
Jennings^145^	w	1000	0	275	78	197
Macdonald^146^	w	191	1	61	30	31
Macdonald et al.^147^	w	56	0	16	5	11
Nemeth et al.^64^	c	827	76	283	134	147
Fanke et al.^148^	w	167	1	24	2	22
Total		2610	81	836	313	521

Avian neoplastic disease prevalence: 81/2610 = 3.1%.

Avian Infectious disease prevalence: 836/2610 = 32.0%.

#### Non-avian reptiles—crocodylians, lepidosaurs and testudines

NARs can develop diseases known from other vertebrate groups, such as mammals or birds^41,74^. Pathological findings in the skeletal system are common, and reports are consistent in mentioning metabolic diseases as a major cause of osseous alterations^41,75^. 90% of orthopaedic cases in NAR veterinary medicine are related to trauma or metabolic disease^41,75^. As previously discussed for birds, metabolic disease or acute trauma can be excluded as differential diagnoses, especially as metabolic diseases are limited to captive animals^4^. NARs demonstrate a wide range of neoplasms^76^, and the overall neoplasm rate in the literature analysis was 6.2% (824/13,299 NARs; Supplementary Table S2). Yet only 3.6%^77^ to 5.5%^78^ of all tumours are located in the bone, resulting in only a small number of neoplasms of the skeletal system^79^. It has to be considered that only a small number of studies have addressed bone involvement in neoplastic diseases overall. Furthermore, there is a lack of systematic histopathological investigations of bone tumours in NARs^77^.

The skeletal tumours that have been identified are the following: osteoma, ameloblastoma, ossified fibroma, osteosarcoma, osteochondroma and chondrosarcoma^40,41,55,77,80^. Studies have shown malignant entities to be dominant among neoplasms in NARs, at approximately 80% of all diagnosed neoplasms (83.3% in Page-Karjian et al., 76% in Ramsay et al.)^81,82^. As osteomas generally appear in long bones^79^ and osteochondromas are defined by articular proximity, these two benign bone tumours are potential differential diagnoses for the lesion of the left fibula and fused vertebrae, respectively. However, these tumours are rarely found in veterinary studies in NARs and in large studies^54,83^, in which several thousand necropsy reports were assessed, no osteomas or osteochondromas were described. Furthermore, the lesions under investigation lack typical features of a benign neoplasm, such as sharp demarcation of the lesion and focal growth. Given the characteristics of the lesions under investigation in this study and the dominance of malignant entities among neoplasms in NARs, we considered a multifocal osseous malignancy as a potential differential diagnosis.

Our analysis of pooled literature data on NAR neoplastic and infectious disease prevalence showed that neoplasms (1.8%, 47/2549 NARs) are distinctly less common than infections (53.9%, 1374/2549 NARs; Table 2, Fig. 6). Specifically, bacterial infections are a major cause of disease among NARs^41,84^, and the prevalence of affected animals in previous studies was as high as 74.1%^85^ or, in a more recent study, 66.5%^41^. Furthermore, bone involvement in the form of osteomyelitis is a common sequela of trauma^75^ and has been described in several studies^9,76,79^. Moreover, Rothschild has shown that bone pathologies unrelated to trauma are only present in less than 0.7% of overall pathologies^42^. The characteristics of osteomyelitis in NARs align with the pathology presented here as reactive and proliferative bone formation, which is commonly observed in NARs with osteomyelitis^41,79^.

**Table 2:** Prevalence of infectious and neoplastic disease in captive non-avian reptiles.

References	Cohort size	Neoplasm	Infection		
			Total	Bacterial	Non-bacterial
Sinn^41^	1941	36	819	536	283
Mendyk et al.^149^	108	6	40	13	27
Ippen^150^	500	5	515	214	301
Total	2549	47	1374	763	611

Non-avian reptile neoplastic disease prevalence: 47/2549 = 1.8%.

Non-avian reptile infectious disease prevalence: 1374/2549 = 53.9%.

#### Non-avian dinosaurs

Generally, diseases are distributed across all major clades of NADs^86^; the most common diseases occur in several species^87^. Fractures and trauma are reported to be the most common pathological conditions among all NAD species^1,88–91^. However, metabolic disorders have also been described, such as Paget’s disease or gout^32,87,92^. Nevertheless, some pathologies are reported predominantly for specific NAD groups^93,94^ (Fig. 6).

##### Neoplasm

Neoplasia is generally rare in vertebrate fossils^86,93^. The earliest record of an NAD neoplasm in form of a haemangioma is documented from an undetermined dinosaur bone fragment of the Upper Jurassic Morrison Formation^95^. Known tumours were limited to hadrosaurs, and the first non-hadrosaurian neoplastic lesions (osteoma and haemangioma) were documented only recently in a Brazilian Late Cretaceous titanosaurid^86^. While metastatic cancer was extremely rare, as shown by a study of more than 10,000 X-rayed specimens^94^, an early metastatic cancer was reported in an undetermined dinosaur bone from the Upper Jurassic Morrison Formation of Colorado^96^. Dumbrava et al. documented the first ameloblastoma in a hadrosaurid dinosaur from the uppermost Cretaceous of Romania^93^. Other data demonstrate a cancer rate in NADs similar to that in extant NARs and birds^97^. In our systematic analysis of the NAD literature, most neoplasms were reported in hadrosaurs; however, some other NAD groups also showed evidence of neoplasms (Fig. 6). Neoplasia has so far not been reported in tyrannosaurids.

##### Infection

The oldest case of bacterial infection (osteomyelitis) was identified in the mandible of a 275-million-year-old captorhinid eureptile *Labidosaurus*^98^. Infected fractures are common in vertebrate fossils^94^, and especially large-bodied theropods are found with fractures, bite marks and infections^88^. Post-traumatic and non-traumatic infections have been reported in many NADs (compiled by Redelstorff et al.^90^), and most bone infections have to be regarded as a complication of trauma^4^. Barbosa et al. recently diagnosed non-specific osteomyelitis in a caudal vertebra of an Aeolosaurini titanosaur^99^; nevertheless, osteomyelitis is poorly documented in NADs^89^ although it has been found in several other fossil reptiles^98,100,101^. Osteomyelitis is often spread from traumatised and infected teeth^93,100^ and develops after injury^98^. Our systematic analysis of the NAD literature revealed that all major clades have been affected by infections (Fig. 6).

##### Trauma and vertebral fusion

Trauma represents the most common pathology in NADs, with fractured and infected ribs being the most common among theropods^88^. Especially tyrannosaurids are known for their frequent skeletal pathologies^18,102^. Possible infected injuries could be reasons for certain disorders^91,103^, as well as their aggressive behaviour—in particular, allosauroids and tyrannosaurids seem to have been involved in aggressive intra- or interspecific biting^15,89,103^. Several studies have reported caudal vertebral pathologies in the fossil record^46,52,99,102,104–107^. The record of pathologies among hadrosaurs shows that damaged and malformed caudal vertebrae are the most common injuries and that intraspecific activities could be the most probable cause^5^. The recently described *Tyrannosaurus rex* RSM P2523.8 also presents with rugosities and deformations of caudal vertebrae; these are still under investigation^102^. The vast number of possible differential diagnoses for fused caudals, including degenerative diseases (although these are extremely rare in the fossil record^108^), and their versatile presentation make the diagnosis particularly difficult^104,106,109^.

### Overall diagnostic consideration

The evaluation of the external and internal morphological features of the fused caudal vertebrae and the left fibula favours the diagnosis of chronic osteomyelitis, demonstrating pathognomonic features such as new bone formation with irregular bone surface, periosteal proliferation and fusion of vertebrae^7,9,37,56,79^. The results of the phylogenetic investigation on disease prevalence demonstrate that infectious disease is substantially more common than neoplastic disease (prevalence: avian infectious disease 32%, neoplasia 3.1%; NAR infectious disease 53.9%, neoplasia 1.8%; Tables 1, 2). In addition, osteomyelitis has also been described in all members of the clade of NADs (Fig. 6), whereas no case of neoplasia has been reported in tyrannosaurids. Therefore, on the basis of (i) evidence from the morphological evaluation of the abnormalities, including the analysis of the internal structure by computed tomography, and (ii) the prevalence of the remaining entities under differential consideration in the NPDB and within the clade of NADs, we conclude that the pathologies of the fused caudal vertebrae and left fibula as described here correspond to chronic osteomyelitis.

## Discussion

This study describes a comprehensive palaeopathological approach for diagnosing disease using morphological analysis and radiological imaging combined with phylogenetic disease bracketing^6,12,28^. This approach was applied to pathological findings of the fused caudal vertebrae and the left fibula of an articulated skeleton of a *Tyrannosaurus rex*, establishing the diagnosis of chronic osteomyelitis with a high level of confidence.

Establishing a definite diagnosis in veterinary medicine, especially in potentially neoplastic diseases, can be challenging and requires radiological imaging, laboratory diagnostics, and often biopsies with subsequent histological work-up^39,40,53^. In human medicine, radiological imaging also plays a crucial role, guiding diagnostic considerations and narrowing down the number of possible differential diagnoses. Specifically, the diagnosis of chronic osteomyelitis should not rely solely on radiography^9,56^, as its appearance in imaging can resemble true osseous neoplasms as well as degenerative diseases^57,110,111^. However, the feasibility of such a diagnostic work-up in palaeopathology is limited. Hence, epidemiology and phylogeny can be used in addition to radiological imaging, providing essential information on possible diseases and their prevalence in order to put differential considerations from imaging into an evolutionary context. The evidence of pathologies found in close relatives in the phylogenetic bracket can thus support a diagnosis in palaeopathology and increase the level of confidence for this diagnosis^6,12,28^.

Despite the considerable knowledge regarding spine-affecting diseases of NADs from several clades^46,52,99,102,104–107,112^, investigative tools in palaeontology remain limited and thus make differential diagnosis challenging. A recent investigation of a fused mid-caudal vertebrae of a titanosaur (CPPLIP-1020) failed to yield a definite diagnosis of either spondyloarthropathy or infection, owing to the lack of distinct morphological features using gross examination and CT imaging, which are considered established techniques in the majority of palaeopathological studies^106^. The present study additionally applied the phylogenetic disease-bracketing approach to assess the likelihood of certain diseases in the taxa investigated. Infectious diseases are considerably more common in birds, NARs, and NADs than are neoplastic diseases, which makes the diagnosis of a neoplastic disease in the pathologies investigated here highly unlikely. Moreover, the complexity of inflammatory processes increases with phylogeny^113^. Therefore, pathomorphological characteristics can differ among species. Although birds and NARs are capable of mounting cellular and humoral immune responses, they do not have an elaborate lymphatic system such as mammals have^113^. Additionally, in contrast to mammals, both NARs and birds deposit fibrin in inflamed and infected areas, leading to the immobilisation of infectious particles^59,88,114^. This fibrin collection (fibriscess) enables the body to keep infections relatively localised, while generalised septicaemia is rarely found^59,88^. However, in turn, the immobilisation of pathogens and leukocytes and the granulomatous cellular reaction commonly lead to chronically persisting infections. The slow continuous growth of the encapsulated focus of infection makes fibriscesses prone to misclassification as neoplasms, e.g. in crocodiles, which can develop fibriscesses in response to bite wounds^59^. The findings of this study also support the hypothesis of fibriscess formation and chronic focal osteomyelitis as opposed to systemic septic spread in the course of advanced osteomyelitis. The two pathological lesions of the *Tyrannosaurus rex* described here are characterised by large locally advanced bone alterations with reactive periosseous formations consistent with morphological features found in fibricesses. Specifically, each of the lesions shows a location of lower bone density and loosened bone texture, indicative of active inflammation, surrounded by newly formed bone of increased density, which encapsulates the lesion focus and prevents systemic dissemination of the pathogen. Furthermore, some chronic avian and NAR infections frequently lead to calcification^113^, which could be an alternative explanation for the hyperdense areas within the pathologically changed bone and which could mimic sediment enclosures. The pathophysiology of chronic infections in birds and NARs with increased intraosseous pressure^4^ can explain the lesion appearance in the *Tyrannosaurus rex* with diffuse thickening of major parts of the bone. Bearing in mind the pathophysiological characteristics of inflammatory processes in birds and NARs, the diffuse expansion of the bones observed here and the imaging features of fistula formation are in accordance with the definition of chronic osteomyelitis^57,58^.

The specific primary formative cause and aetiopathology of the chronic infection in the affected bones here remains unknown. It has been shown that in theropods the tail is the body part most susceptible to injury^115^, and several hadrosaurs with traumatic changes of the tail have been reported^52,116^. Possible causes of osteomyelitis in NARs and birds include bone exposure and trauma^62,75,79,117^, which is the most common cause of osteomyelitis in birds^38^. In the light of the fact that traces of head-biting behaviour have particularly been observed among tyrannosaurids it can be assumed that the bone infection, in particular the vertebral pathology, originates from a traumatic event^103,118^. Furthermore, it remains unclear whether the diagnosis of chronic osteomyelitis in the caudal vertebrae and left fibula caused the death of the individual and whether the two lesions occurred simultaneously or consecutively. However, it is likely that the advanced stage of these pathologies had an impact on locomotion, because of mechanical alterations related to swelling or because of pain. As haematogenous osteomyelitis mostly involves the long bones (particularly tibia and fibula) and vertebral osteomyelitis is most frequently of the haematogenous type^56^, the death of the specimen investigated here could have been the result of a systemic septic process in the course of several advanced chronic bone infections. Moreover, chronic osteomyelitis is commonly subclassified into primary or secondary chronic osteomyelitis. However, subclassification of osteomyelitis in this study is not applicable, since the criteria for distinguishing between primary and secondary disease include primary aetiology as well as duration^119, 120^. Nonetheless, the morphological characteristics of the lesions as described above suggest that each lesion of chronic osteomyelitis comprised an active focus of infection with lowered bone density and loosened bone texture.

FMNH PR2081 exhibits numerous skeletal pathologies beside those examined herein, including lesions in the jaw^26^, on the right forelimb^28,32^, on several ribs^26,28^, and in the proximal caudal neural spines^26,28^. These have been the topic of several other studies, and a review of the full medical history revealed by these injuries is well beyond the scope of this report. Suffice it to say that these injuries, taken together, indicate that FMNH PR2081 experienced a plethora of injuries and diseases over the course of its lifespan. In this respect, it can be informative to infer when in life these pathologies occurred, to understand how they might have affected the biology of this exemplary species. In particular, the two pathologies examined here appear to have developed after the individual had reached adult size, as revealed by the ‘phantom’ outlines of the original caudal centra and fibula shaft visible in the CT images. Histological ageing of FMNH PR2081, based on several findings, has determined that this individual was 28–33 years old at the time of death^20,121^, and that it had reached growth asymptote roughly a decade earlier. Thus, the likely osteomyelitis observed in the caudals and fibula appears to have afflicted the animal for less than a decade after somatic maturity was attained. Osteomyelitis has also been documented in a younger individual^101^ referred to (by some) as *Tyrannosaurus rex* and histologically aged as around 12 years old^122^, so osteomyelitis could afflict *Tyrannosaurus rex* throughout its life.

This study has some limitations. The pathology reports on birds and NARs are based mainly on individuals held in captivity, which implies medical care and a somewhat artificial environment, resulting in a prolonged lifespan. The latter inherently entails the risk of higher incidence rates of neoplasms, and this may in turn have affected the calculation of the respective disease prevalence. This is emphasized by the fact that neoplasms in free-flying birds are extremely rare. In addition, the gold standard for palaeopathology is the histological examination of specimens, and museum policies did not permit invasive diagnostics in the case of the two pathological lesions described. Furthermore, cited studies on diseases in paleontology differed in their methodological approach, of which a few used EPB to reach their diagnosis^12,13,15,88,123,124^.

In conclusion, this study presents a comprehensive diagnostic approach tailored to palaeopathology. Given the relatively small amount of diagnostic information preserved in fossilised material, essential and well-established detailed morphological analysis including radiological imaging has been combined with epidemiological studies through phylogenetic disease-bracketing. This introduced two lines of evidence for the diagnosis of chronic osteomyelitis in the fused caudal vertebrae and left fibula of a *Tyrannosaurus rex*. This study provides evidence supporting the use of epidemiological data within the clade of NADs and its NPDB as a reference for further palaeopathological studies.

## Methods

### The fossil specimen

The subject of this study was the *Tyrannosaurus rex* specimen FMNH PR2081 from the Maastrichtian (Late Cretaceous; approximately 67 million years old), nicknamed ‘Sue’ and is housed at the Field Museum of Natural History in Chicago, IL, USA. The specimen was excavated from mudstones of the lower Hell Creek Formation, near Faith in South Dakota, and is one of the best preserved and most complete *Tyrannosaurus rex* skeletons known. About 70% of the skeleton was recovered in 1991 by the Black Hills Institute of Geological Research, Inc. following its discovery by fossil-hunter Sue Hendrickson. It is now on permanent display at the Field Museum. Previous research has focused on several potentially pathological findings of this skeleton^15,26,27^. In this study the pathological lesions of the left fibula and fused vertebrae c26 and c27 were investigated (Fig. 1). The fibula is 104 cm long and its largest diameter, at the level of the pathological bone alteration, is 16 cm (Fig. 1). The rostral-caudal dimension of the fused vertebral bodies is 26.5 cm and their mediolateral dimension is 19.8 cm (Fig. 1).

### Evaluation of disease based on morphology—CT imaging

In order to investigate the abnormality, apart from visual inspection and surveying, a medical 16-slice CT scanner (Bright Speed, GE Medical Systems, Waukesha, WI, USA) was used for radiological imaging of the internal structure of this large fossil specimen. This CT scanner has a large gantry opening and is thus suitable for this large fossil. The following scanning protocol was used: collimation 16 × 0.625 mm, 120 kV, tube current 440 & 305 mA. For reconstruction, a soft-tissue and bone kernel with an image thickness of 0.625 mm for the caudals and an image thickness of 1.25 mm for the left fibula were used. Multiplanar reconstructions were generated by using dedicated post-processing software.

### Evaluation of disease based on phylogeny

In order to incorporate the likelihood of respective diseases in NADs (and specifically in *Tyrannosaurus rex*) for establishing the diagnosis, evidence on the prevalence of diseases was extracted systematically from the literature and the relevant information was pooled. The phylogeny of NADs is currently under debate, pending a possible re-sorting of the major clades^125^. For this study, a more “conservative” phylogenetic tree for NADs was used, which was recently corroborated by Langer et al.^126^. This phylogeny uses the “traditional” major NAD clades Ornithischia and Saurischia. The phylogenetic tree includes only those groups in which neoplastic and infectious diseases are known. Tree topology is based on Benson et al.^127^.

The narrow phylogenetic framework for NADs, with brackets including birds and NARs, was used to map the diseases onto the single clades of NADs. Veterinary literature on diseases in extant birds and NARs was searched for pathological reports, and the prevalence of the respective diseases was included in the analysis. In order to calculate disease prevalence, and to avoid reporting bias, only studies were included in which the prevalence of infectious and neoplastic disease within the same cohort was investigated (Tables 1 and 2). To assess further the rate of neoplasia in extant birds and NARs, an analysis of pooled studies reporting the number of neoplasms in the respective animal cohorts was performed and overall avian and NAR neoplasm rates were calculated (see Supplementary Tables S1 and S2). To investigate all possible differential diagnoses, given the enormous knowledge on human as compared with veterinary diseases, literature on human diseases was also reviewed. Literature on diseases in NADs was searched for pathology reports and descriptions of osseous lesions. The literature search was focused on tumorous growth and on neoplastic and infectious lesions in the above-mentioned species; it was conducted in the following databases: Google Scholar, PubMed, Web of Science and Scopus. The databases were searched with the following mesh terms under the following constellations: ((Bird* OR Avian OR Reptile*) AND (neoplasm* OR tumor* OR infect* OR inflammat*)); ((dinosaur* OR saurischian OR tyrannosaurus) AND (neoplasm* OR tumor* OR infect* OR inflammat*)). Common textbooks were also reviewed that were available at the local veterinary library and the local medical library. Literature published up to March 2019 was included in the searches.

## Supplementary information

Supplementary Information

## Data Availability

The specimen investigated is housed in the Field Museum of Natural History, Chicago, IL, USA under the collection number FMNH PR2081. Access to the CT images is provided by the Field Museum of Natural History via https://www.fieldmuseum.org/field-museum-natural-history-conditions-and-suggested-norms-use-collections-data-and-images.
