# Supplementary material for: A comprehensive diagnostic approach combining phylogenetic disease bracketing and CT imaging reveals osteomyelitis in a Tyrannosaurus rex
Source: Sci Rep. 2020 Nov 3;10:18897. doi: 10.1038/s41598-020-75731-0 (PMC7642268; doi:10.1038/s41598-020-75731-0)
Supplement: Supplementary file 1 — Supplementary Information [file 41598_2020_75731_MOESM1_ESM.docx]

**A comprehensive diagnostic approach combining phylogenetic disease bracketing and CT imaging reveals osteomyelitis in a *Tyrannosaurus rex***

C.A. Hamm^1,2^, O. Hampe^3^, D. Schwarz^3^, F. Witzmann^3^, P. Makovicky^4,5^, C. Brochu^6^, R. Reiter^1,7^, P. Asbach^1^*

^1^Department of Radiology, Charité – Universitätsmedizin Berlin, corporate member of Freie Universität Berlin, Humboldt-Universität zu Berlin and Berlin Institute of Health, 10117 Berlin, Germany

^2^Department of Radiology, Pomeranian Medical University, 70-204 Szczecin, Poland

^3^Museum für Naturkunde, Leibniz-Institut für Evolutions- und Biodiversitätsforschung, Invalidenstraße 43, 10115 Berlin, Germany

^4^Field Museum of Natural History, 1400 S. Lake Shore Dr., Chicago, IL 60605, USA

^5^Department of Earth and Environmental Sciences, University of Minnesota, Minneapolis, MN 55455, USA

^6^Department of Earth & Environmental Sciences, University of Iowa, Iowa City, IA 52242, USA

^7^Richard and Loan Hill Department of Bioengineering, University of Illinois at Chicago, Chicago, IL 60607, USA

**Address for correspondence**

Patrick Asbach, MD

Charité – Universitätsmedizin Berlin

Dept. of Radiology

Charitéplatz 1, Berlin 10117, Germany

phone: +49 30 450 527723

fax: +49 450 7527953

email: [patrick.asbach@charite.de](mailto:patrick.asbach@charite.de)

ORCID ID: 0000-0002-6885-3283

**Supplemental information**

**Supplementary Table S1.** Overall neoplasia disease rate in birds

| **Reference** | **Wild (w) or captive (c)** | **Cohort** | **Neoplasm** |
| --- | --- | --- | --- |
| **Dillberger et al. 1987 ^1^** | c | 127 | 4 |
| **Loupal & Reifinger 1986 ^2^** | w & c | 6211 | 150 |
| **Macneill & Barnard 1978 ^3^** | c | 174 | 1 |
| **Macneill & Barnard 1978 ^3^** | w | 636 | 0 |
| **Reece 1992 ^4^** | w & c | 10000 | 383 |
| **Shimonohara et al. 2013 ^5^** | w | 83 | 28 |
| **Siegfried 1983 ^6^** | w | 18000 | 9 |
| **Sokkar et al. 1979 ^7^** | c | 1248 | 126 |
| **Hubbard et al. 1983 ^8^** | c | 475 | 17 |
| **Effron et al. 1977 ^9^** | c | 5957 | 111 |
| **Chu et al. 2012 ^10^** | c | 873 | 37 |
| **Reece 1996 ^11^** | c | 90000 | 2281 |
| **Lombard & Witte 1959 ^12^** | c | 10240 | 138 |
| **Santos et al. 2008 ^13^** | w | 253 | 13 |
| Total |  | 144277 | 3298 |
| Overall avian neoplasia disease rate: 3298/144277= 2.3% | | | |

**Supplementary Table S2.** Neoplasia disease rate in non-avian reptiles overall and the non-avian reptile taxon Lepidosauria, Testudines, and Crocodylia.

| **Reference** | **Wild (w) or captive (c)** | **Cohort** | **Neoplasm** |  |  |  |
| --- | --- | --- | --- | --- | --- | --- |
| Studies without cohort information regarding reptile taxons | | | |  |  |  |
| **Chu et al. 2012**^#^ **^10^** | c | 449 | 5 |  |  |  |
| **Effron et al. 1977**^#^ **^9^** | c | 1233 | 28 |  |  |  |
| **Hubbard et al. 1983**^#^ **^8^** | c | 143 | 5 |  |  |  |
| Studies with specified cohort information regarding reptile taxons | | | | **Taxon** | **Specified cohort** | **Neoplasm** |
| **Garner et al. 2004 ^14^** | n/a | 5353 | 527 |  |  |  |
|  | | | | Lepidosauria | 4095 | 488 |
|  |  |  |  | Testudines | 1067 | 29 |
|  |  |  |  | Crocodylia | 185 | 4 |
|  |  |  |  | Unknown | 6 | 6 |
| **Hernandez-Divers & Garner 2003 ^15^** | n/a | 1297 | 81 |  | | |
|  | | | | Lepidosauria | 1297 | 81 |
| **Page-Karjian et al. 2017 ^16^** | c | 255 | 37 |  | | |
|  | | | | Lepidosauria | 255 | 37 |
| **Ramsay et al. 1996 ^17^** | c | 65 | 20 |  | | |
|  | | | | Lepidosauria | 65 | 20 |
| **Sykes & Trupkiewicz 2006 ^18^** | c | 3684 | 78 |  | | |
|  | | | | Lepidosauria | 3084 | 72 |
|  |  |  |  | Testudines | 511 | 6 |
|  |  |  |  | Crocodylia | 89 | 0 |
| **Catao-Dias & Nichols 1999 ^19^** | c | 291 | 36 |  | | |
|  | | | | Lepidosauria | 291 | 36 |
| **Schmidt-Ukaj et al. 2017 ^20^** | c | 529 | 7 |  | | |
|  | | | | Lepidosauria | 529 | 7 |
| Total |  | 13299 | 824 |  | | |
| Overall non-avian reptile neoplasia disease rate: 824/13299 = 6.2%  Overall Lepidosauria neoplasia disease rate: 741/9616 = 7,7%  Overall Testudines neoplasia disease rate: 35/1578 = 2,2%  Overall Crocodylia neoplasia disease rate: 4/274 = 1,5% | | | |  |  |  |

^#^ these studies did not specify the taxon of investigated non-avian reptiles

**Supplementary references**

1 Dillberger, J. E., Citino, S. B. & Altman, N. H. Four cases of neoplasia in captive wild birds. *Avian Dis* **31**, 206-213 (1987).

2 Loupal, G. & Reifinger, M. Tumors in birds in zoos, in the wild and kept as companions. *Journal of Veterinary Medicine Series a-Zentralblatt Fur Veterinarmedizin Reihe a-Physiology Pathology Clinical Medicine* **33**, 180-192 (1986).

3 Macneill, A. C. & Barnard, T. Necropsy results in free-flying and captive Anatidae in British Columbia. *Can Vet J* **19**, 17-21 (1978).

4 Reece, R. L. Observations on naturally occurring neoplasms in birds in the state of Victoria, Australia. *Avian Pathol* **21**, 3-32, doi:10.1080/03079459208418815 (1992).

5 Shimonohara, N., Holland, C. H., Lin, T. L. & Wigle, W. L. Naturally occurring neoplasms in pigeons in a research colony: a retrospective study. *Avian Dis* **57**, 133-139, doi:10.1637/10244-051012-Case.1 (2013).

6 Siegfried, L. M. Neoplasms identified in free-flying birds. *Avian Dis* **27**, 86-99 (1983).

7 Sokkar, S. M., Mohammed, M. A., Zubaidy, A. J. & Mutalib, A. Study of some non-leukotic avian neoplasms. *Avian Pathol* **8**, 69-75, doi:10.1080/03079457908418328 (1979).

8 Hubbard, G., Schmidt, R. & Fletcher, K. Neoplasia in zoo animals. *The Journal of Zoo Animal Medicine* **14**, 33-40 (1983).

9 Effron, M., Griner, L. & Benirschke, K. Nature and rate of neoplasia found in captive wild mammals, birds, and reptiles at necropsy. *JNCI: Journal of the National Cancer Institute* **59**, 185-198, doi:10.1093/jnci/59.1.185 (1977).

10 Chu, P. Y. *et al.* Spontaneous neoplasms in zoo mammals, birds, and reptiles in Taiwan - a 10-year survey. *Animal Biology* **62**, 95-110, doi:10.1163/157075611x616941 (2012).

11 Reece, R. Some observations on naturally occurring neoplasms of domestic fowls in the State of Victoria, Australia (1977-87). *Avian pathology: journal of the WVPA* **25**, 407 (1996).

12 LOMBARD, L. & WITTE, E. Frequency and types of tumors in mammals and birds of the Philadelphia Zoological Garden. *Cancer research* **19**, 127 (1959).

13 Santos, G. G. C. *et al.* Diseases of wild birds diagnosed at the Federal University of Parana, Brazil (2003-2007). *Pesquisa Veterinaria Brasileira* **28**, 565-570 (2008).

14 Garner, M. M., Hernandez-Divers, S. M. & Raymond, J. T. Reptile neoplasia: A retrospective study of case submissions to a specialty diagnostic service. *Vet. Clin. North Am. Exot. Anim. Pract.* **7**, 653-671, doi:10.1016/j.cvex.2004.04.002 (2004).

15 Hernandez-Divers, S. M. & Garner, M. M. Neoplasia of reptiles with an emphasis on lizards. *Vet Clin North Am Exot Anim Pract* **6**, 251-273 (2003).

16 Page-Karjian, A. *et al.* NEOPLASIA IN SNAKES AT ZOO ATLANTA DURING 1992-2012. *Journal of Zoo and Wildlife Medicine* **48**, 521-524 (2017).

17 Ramsay, E. C., Munson, L., Lowenstine, L. & Fowler, M. E. A retrospective study of neoplasia in a collection of captive snakes. *Journal of Zoo and Wildlife Medicine* **27**, 28-34 (1996).

18 Sykes, J. M. & Trupkiewicz, J. G. Reptile neoplasia at the Philadelphia Zoological Garden, 1901-2002. *Journal of Zoo and Wildlife Medicine* **37**, 11-19, doi:10.1638/04-112.1 (2006).

19 Catao-Dias, J. & Nichols, D. Neoplasia in snakes at the national zoological park, Washington, DC (1978–1997). *Journal of comparative pathology* **120**, 89-95 (1999).

20 Schmidt-Ukaj, S. *et al.* A survey of diseases in captive bearded dragons: A retrospective study of 529 patients. *Veterinarni Medicina* **62**, 508-515, doi:10.17221/162/2016-VETMED (2017).
